# Editorial: New methodological, interventions and neuroscientific perspectives in sport psychology

**DOI:** 10.3389/fpsyg.2022.1061236

**Published:** 2022-12-16

**Authors:** Antonio Hernández-Mendo, M. Teresa Anguera, Verónica Morales-Sánchez, José María Caramés Tejedor

**Affiliations:** ^1^Faculty of Psychology, University of Málaga, Málaga, Spain; ^2^Faculty of Psychology, Institute of Neurosciences, University of Barcelona, Barcelona, Spain; ^3^Centre for the Discovery of Brain Sciences, University of Edinburgh, Edinburgh, United Kingdom

**Keywords:** sport psychology, methodology, mixed methods, intervention, neurosciences

This Research Topic introduces selected articles on sport studies, mainly focused on methodological, interventional, and neuroscientific perspectives, that provide the basis for the title of the current volume. All of these topics are represented herein and we believe that our readers will value this balanced framework.

With the aim of providing a panoramic view of the 27 articles that form this Research Topic, and properly assessing the contributions made, we present a transversal perspective from the following criteria:

1. Conceptual: Although most of the 27 articles are “original research,” with only one wholly fitting into the conceptual category, it is beyond any doubt that numerous articles contained herein highlight the conceptual implications of their research. This Research Topic includes some reference works focused on the self-determination theory, including the mini-theory of basic psychological needs (Tristán et al.; Valenzuela et al.; Vergara-Torres et al.), the achievement goal theory (Martínez-González et al.; Pineda-Espejel et al.), mental toughness (Moreira et al.), the self-efficacy theory (De La Cruz et al.; Monteiro et al.), ecological momentary assessment (González-Barato et al.), and mixed methods (Izquierdo and Anguera).

2. Methodological: For this criterion, a variety of procedural orientations are differentiated in the empirical studies contained herein. Some of the studies use selective procedures (Bracero-Malagón, et al.; De La Cruz et al.;  López-Aymes et al.; Martínez-González et al.; Moreira et al.; Pineda-Espejel et al.; Tristán et al.; Valenzuela et al.; Vergara-Torres et al.), some have a psychometric special interest (Cid et al.; Monteiro et al.), some are observational (Fernandes et al.; Godoy-Izquierdo et al.; Granda-Vera et al.; Menescardi et al.; Nunes et al.; Pastrana-Brincones et al.; Prudente et al.), some are experimental/quasi-experimental (Cui et al.; De La Vega et al.; Hong et al.), and others apply a combination of the preceding ones, using multimethod studies that employ, for instance, observational and selective methodology (Escolano-Perez, et al.; Rodrigues et al.).

Among empirical studies, especially in those that use observational methodology, there is a clear interest in mixed methods, which have acquired so much relevance in recent years due to an eagerness to integrate qualitative and quantitative approaches. In some articles, the mixed method perspective is adopted, either in an explicit or implicit way (Escolano-Pérez et al.; Granda-Vera et al.; López-Aymes et al.; Nunes et al.; Pastrana-Brincones et al.; Rodrigues et al.). The majority of the instruments used consisted of questionnaires (Cid et al.; De La Cruz et al.; Godoy-Izquierdo and Díaz; López-Aymes et al.; Martínez-González et al.; Moreira et al.; Pineda-Espejel et al.; Rodrigues et al.), scales (De La Cruz et al.;  Monteiro et al.; Tristán et al.; Valenzuela et al.; Vergara-Torres et al.), inventories (Escolano-Pérez et al.), and even tests (Bracero-Malagón et al.), although *ad hoc* instruments have been built for observational studies (Escolano-Pérez et al.; Fernandes et al.; Granda-Vera et al.; Menescardi et al.; Nunes et al.; Pastrana-Brincones et al.; Rodrigues et al.). Additionally, some studies used structural and functional magnetic resonance images (Cui et al.).

Moreover, the different types of software used in the studies featured in this Research Topic can be of interest to readers. Such software includes programs such as ALCESTE (López-Aymes et al.), AMOS (Monteiro et al.; Tristán et al.), AQUAD (Granda-Vera et al.), GSEQ5 (Nunes et al.), GRETNA (Cui et al.), HOISAN (Granda-Vera et al.; Menescardi et al.; Pastrana-Brincones et al.), LINCE PLUS (Fernandes et al.), SPSS (Bracero-Malagón et al.; Escolano-Pérez et al.; González-Barato et al.; López-Aymes et al.; Martínez-González et al.; Pineda-Espejel et al.; Rodrigues et al.), and THEME (Fernandes et al.). In particular, we highlight the new software “Function Estimation”, contained in the MENPAS platform and described by Pastrana-Brincones et al..

A diverse range of data analyses are featured in this Research Topic, including content analysis (López-Aymes et al.), cluster analysis (Valenzuela et al.), polar coordinates analysis (Granda-Vera et al.; Menescardi et al.; Nunes et al.; Pastrana-Brincones et al.), analysis of structural equations (De La Cruz et al.; Martínez-González et al.; Monteiro et al.; Pineda-Espejel et al.; Tristán et al.; Vergara-Torres et al.), factorial analysis (Cid et al.; De La Cruz et al.; Moreira et al.; Pineda-Espejel et al.; Vergara-Torres et al.), linear regression analysis (Bracero-Malagón et al.), logistic regression analysis (De La Vega et al.), sequential delay analysis (Menescardi et al.), ANCOVA (Hong et al.), ANOVA (Cui et al.; Escolano-Pérez et al.), T-Patterns detection (Fernandes et al.), MANOVA (López-Aymes et al.), and temporal series (González-Barato et al.). In the observational studies data, quality control was applied before the data analysis, using either consensual concordance (Escolano-Pérez et al.), the kappa coefficient (Granda-Vera et al.; Nunes et al.; Rodrigues et al.), or generalizability theory (Bracero-Malagón et al.; Pastrana-Brincones et al.; Rodrigues et al.).

3. Technological: we would like to highlight a Brief Research Report describing the development of the B alert (De La Vega et al.) and PSIXPORT (González-Barato et al.) apps.

4. Scope of application: All the articles published in this Research Topic belong in the area of sport psychology, although many of them focus on specific subjects, such as physical activity (López-Aymes et al.; Valenzuela et al.), athletes (Martínez-González et al.; Moreira et al.), basketball (Granda-Vera et al.; Nunes et al.; Pastrana-Brincones et al.), quality of life (López-Aymes et al.), team coordination (Fernandes et al.), elite sports (Monteiro et al.; Pineda-Espejel et al.), aerobic exercises (Cui et al.), coaches and training (Limballe et al.; Rodrigues et al.), fatigue (De La Vega et al.), fitness (Bracero-Malagón et al.), soccer (Godoy-Izquierdo and Díaz; Monteiro et al.; Tristán et al.), sports injuries (González-Barato et al.), taekwondo (Menescardi et al.), and motor skills/task (Escolano-Pérez et al.; Hong et al.). Additionally, some reviews compile up-to-date knowledge on perceptual aspects of sports training (Limballe et al.), hypnosis (Li and Li), and the translation and validation of questionnaires (Cid et al.).

In short, with our best wishes, 27 articles that will help to expand the knowledge of our readers are presented in this Research Topic.

## Conclusions

Articles featured in this Research Topic offer a wide spectrum of theoretical and methodological contributions, and as editors, we would like to express our satisfaction with the materialization of these works and their transfer to the scientific community.

The articles that make up this Research Topic already have a high number of “views” according to inner metrics. All articles were published between 2021 and 2022. Of the 30 articles that were initially submitted, 27 were accepted, resulting in an acceptance percentage of 90%.

## Author contributions

AH-M coordinated the editorial work. MTA, JC, and VM-S contributed to the process. MTA wrote the article for this editorial letter. JC, AH-M, and VM-S reviewed the final version. All authors contributed to the article and approved the submitted version.

